# Supplementary material for: A conformational change in α-catenin’s actin-binding domain governs adherens junction maturation
Source: Commun Biol. 2025 Sep 1;8:1325. doi: 10.1038/s42003-025-08785-3 (PMC12402239; doi:10.1038/s42003-025-08785-3)
Supplement: Supplementary file 2 — Supplementary Information [file 42003_2025_8785_MOESM2_ESM.pdf]

Supplementary Information

for

A conformational change in α-catenin's actin-binding domain governs adherens junction maturation

Lukas Windgasse and Carsten Grashoff

Correspondence to C.G. (e-mail: grashoff@uni-muenster.de)

This pdf file includes:

Supplementary Figs. 1-7 and Supplementary Figure Legends

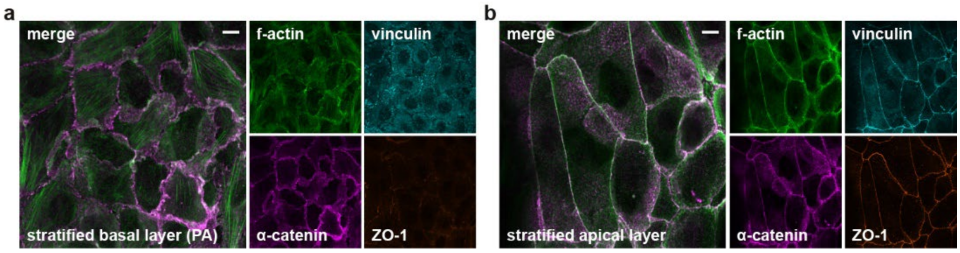

Supplementary Figure 1 – Protein localizations in stratifying MEK cultures.

a, **b**, Representative images of differentiated MEKs immunostained after prolonged cell culture and stratification. F-actin (green), α-catenin (magenta), vinculin (turquoise) and ZO-1 (orange). The merged images show the signal from the f-actin and α-catenin channels. **a**, The basal cell layer of stratified MEKs is characterized by presence of actin stress fibers and vinculin signals in focal adhesions. ZO-1 is barely detectable in these areas. **b**, The top layer of stratified cell cultures displays enlarged cells with distinct cell-cell junctions. F-actin displays a cortical localization; α-catenin, vinculin and ZO-1 are colocalized at cell-cell junctions. Scale bar: 10 µm.

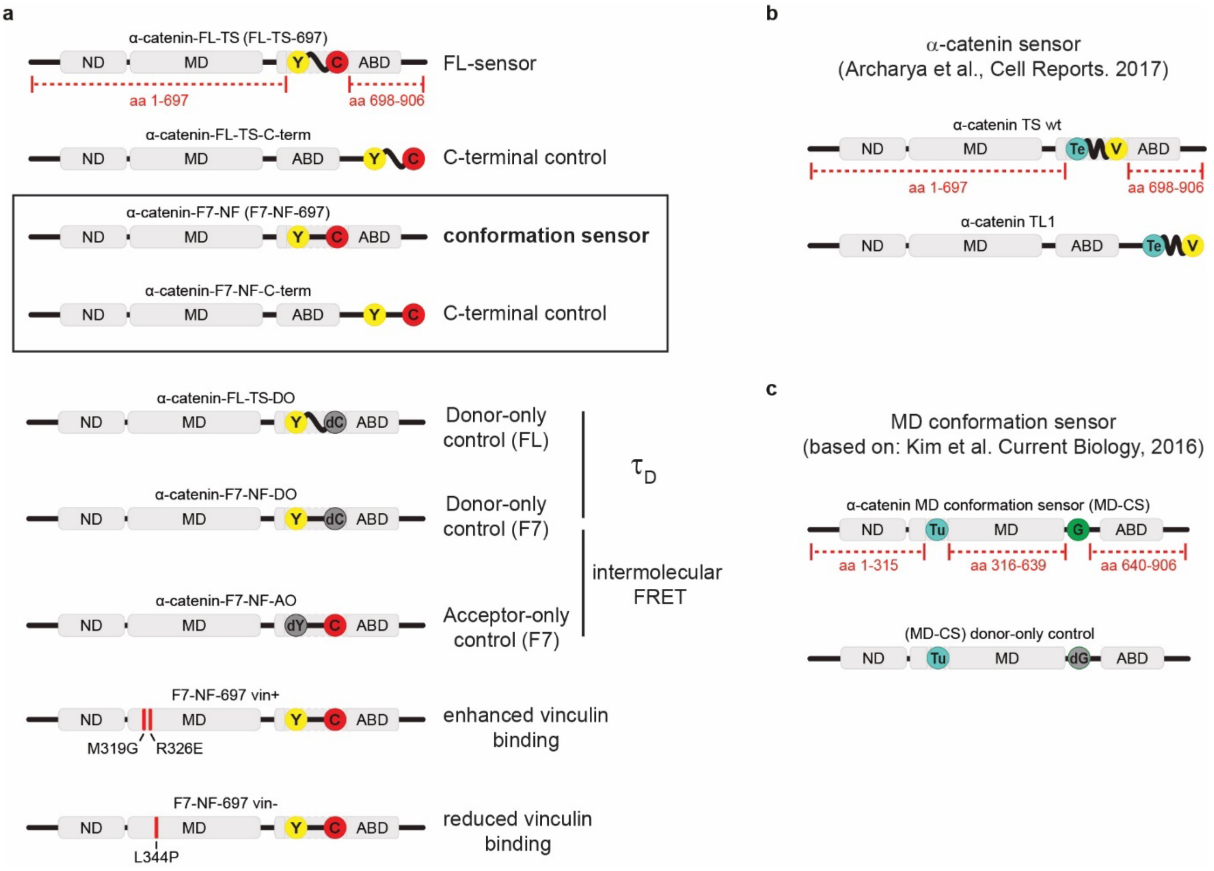

Supplementary Figure 2 – Overview of the here generated and previously reported α-catenin expression constructs.

a, Schematic depiction of α-catenin expression constructs generated in this study. Distinct FRET modules, using YPet (Y) as the donor and mCherry (C) as the acceptor, were inserted at the N-terminal part of the actin-binding domain (ABD) after aa 697. Insertion of the force-insensitive F7-no force (NF) module yields the here used conformation sensor (F7-NF-697); fusion of the same module to the C-terminal end of α-catenin yields the C-terminal control (F7-NF-C-term). To determine the donor lifetime (τ_D) and the calculation of FRET efficiencies, we generated donor-only (DO) constructs by point mutating the mCherry chromophore. To facilitate intermolecular FRET measurements, an acceptor-only (AO) constructs was generated by point mutating the YPet chromophore. α-catenin mutants to evaluate the role of vinculin binding were created by inserting point mutations, i.e., M319G/R326E for enhanced and L344P for reduced vinculin engagement. **b**, Schematic depiction of the previously reported tension sensor constructs, which used the mTFP (Te)–Venus (V) FRET pair that was inserted at aa 697. **c**, Schematic depiction of the here recreated α-catenin conformation sensor,

in which the donor fluorophore was inserted into the middle domain (MD) after aa 315, and the acceptor fluorophore was inserted between the MD and ABD after aa 639. In our study, mTurquoise2 (TU) was used as the donor and ShadowG (G) as the acceptor fluorophore. A donor-only control to calculate FRET efficiencies was established by point-mutating ShadowG at Y67G (dG).

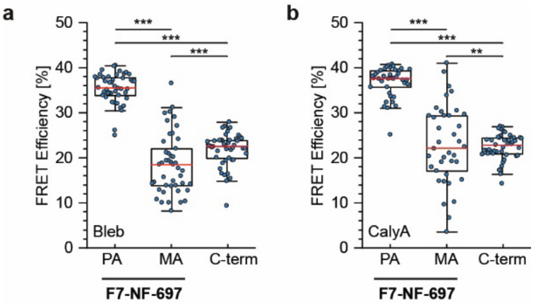

Supplementary Figure 3 – The conformation sensor is insensitive to acute changes in actomyosin tension.

a, Differentiated MEKs expressing the F7-NF-697 conformation sensor or the C-terminal control (C-term) were treated with 50 µM para-amino-blebbistatin for 1 h and analyzed in FLIM-FRET experiments. FRET efficiency differences between PA and MA are maintained. C-terminal controls display a narrow range of FRET efficiencies at around 20 %. (N=3 replicates, n=45, 45, 45). **b**, Differentiated MEKs expressing the F7-NF-697 conformation sensor or the C-terminal control (C-term) were treated with 10 nM Calyculin-A (CalyA) for 1 h. Also under these circumstances, FRET efficiencies are high in PA and low in MA, while C-terminal controls display an unaltered, narrow range of FRET efficiencies at around 20 %. (N=3 replicates, n=43, 39, 38). Two-sample KS test: ** $p<0.01$, *** $p<0.001$. Boxplots are showing the median, the 25th and 75th percentile and whiskers reaching to the last data point within 1.5× interquartile range.

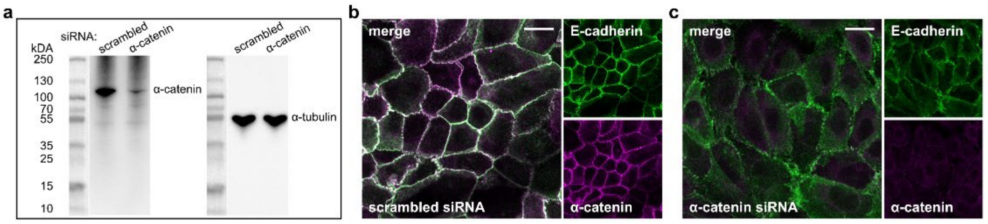

Supplementary Figure 4 – The siRNA mediated knockdown efficiently reduces α-catenin protein levels.

a, Western blot analysis of siRNA-treated MEKs reveals efficient knockdown (KD) of α-catenin. **b**, **c**, Representative images of differentiated MEKs treated with either a scrambled siRNA control (b) or an α-catenin-specific siRNA targeting the 3'-UTR (c). Cells were labeled for E-cadherin (green) and α-catenin (magenta). Note the lack of α-catenin signal in cells treated with α-catenin-specific siRNA. Scale bar: 10 µm.

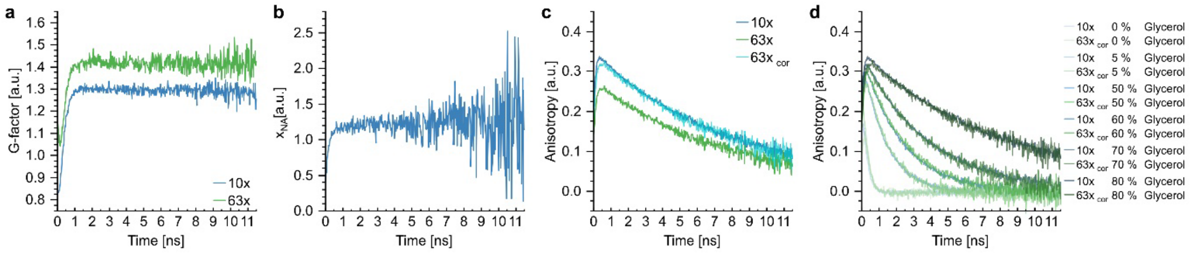

Supplementary Figure 5 – Calibration of the fluorescence anisotropy measurements.

a, Time-resolved variation of the G-factor for the 10× and 63× objectives in the setup used here. **b**, Time-resolved variation of x_{NA} calibration factor according to[25] for the 63× objective. **c**, Time-resolved anisotropy data of an ATTO 514 solution (5 µM) containing 80 % of glycerol measured with 10× and 63× objectives, shown together with correction according to[25] (63×$_{cor}$). **d**, Time-resolved anisotropy of ATTO 514 solutions of increasing glycerol concentration measured with a 10× objective and a 63× objective (corrected).

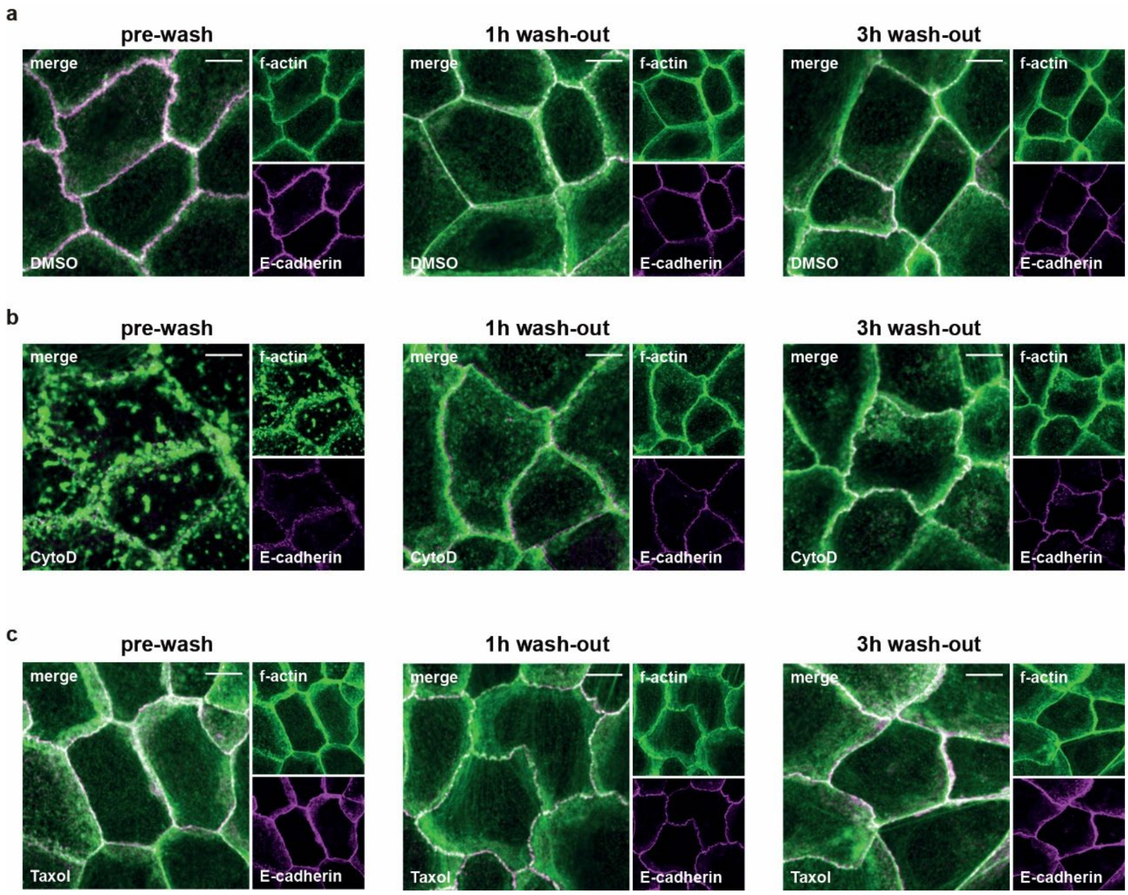

Supplementary Figure 6 – Wash-out control experiments.

a, Representative immunostainings of differentiated MEKs during DMSO treatment and after wash-out. Cells were labeled for f-actin (green) and E-cadherin (magenta). At the here used concentrations, DMSO does not visibly affect cell-cell junctions at the used concentrations. **b**, Representative immunostainings of differentiated MEKs under CytoD treatment (pre-wash), 1 h and 3 h after CytoD wash-out. Note that the cortical f-actin is quickly re-established when CytoD is removed. **c**, Representative immunostainings of differentiated MEKs under Taxol treatment (pre-wash), 1 h and 3 h after Taxol wash-out. The cortical localization of f-actin is maintained after wash-out. Scale bars: 10 µm.

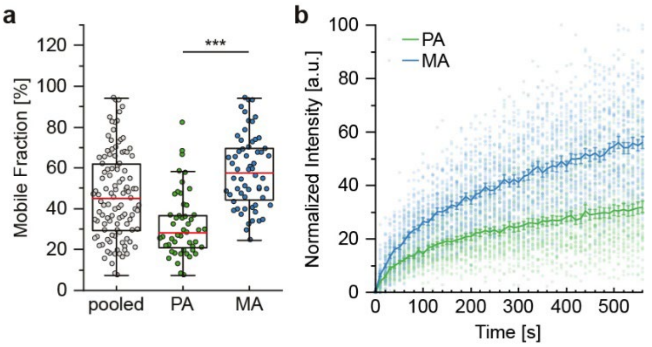

Supplementary Figure 7 – FLIM-FRET/FRAP control experiments.

a, Recovery of the normalized mean fluorescence intensities from FRAP experiments in cells expressing F7-NF-697 in which the acceptor fluorophore mCherry was rendered non-fluorescent by a point-mutation (Y72L); data are categorized into PA (green) and MA (blue). Similar to the experiments shown in Fig. 5c, the intensities in PA recover with different dynamics as compared to MA. Boxplots are showing the median, the 25th and 75th percentile and whiskers reaching to the last data point within 1.5× interquartile range. Two-sample KS test: *** $p<0.001$. **b**, Consistent with Fig. 5d, the calculated mobile fractions indicate a more efficient protein turnover of α-catenin molecules in MA, whereas low mobile fractions are associated with PA. Recovery of the normalized mean fluorescence intensities are shown with s.e.m.
