# Supplementary material for: A conformational change in α-catenin’s actin-binding domain governs adherens junction maturation
Source: Commun Biol. 2025 Sep 1;8:1325. doi: 10.1038/s42003-025-08785-3 (PMC12402239; doi:10.1038/s42003-025-08785-3)
Supplement: Supplementary file 3 — Description of Additional Supplementary Files [file 42003_2025_8785_MOESM3_ESM.pdf]

Description of Additional Supplementary Files

File name: Supplementary Data 1

Description: Source Data to all data sets in Main and Supplementary Figures
