# Supplementary material for: A conformational change in α-catenin’s actin-binding domain governs adherens junction maturation
Source: Commun Biol. 2025 Sep 1;8:1325. doi: 10.1038/s42003-025-08785-3 (PMC12402239; doi:10.1038/s42003-025-08785-3)
Supplement: Supplementary file 5 — Reporting Summary [file 42003_2025_8785_MOESM5_ESM.pdf]

nature portfolio

Corresponding author(s): Carsten Grashoff

Last updated by author(s): Jul 4, 2025

Reporting Summary

Nature Portfolio wishes to improve the reproducibility of the work that we publish. This form provides structure for consistency and transparency in reporting. For further information on Nature Portfolio policies, see our Editorial Policies and the Editorial Policy Checklist.

Statistics

For all statistical analyses, confirm that the following items are present in the figure legend, table legend, main text, or Methods section.

n/a	Confirmed	
☐	☒	The exact sample size (*n*) for each experimental group/condition, given as a discrete number and unit of measurement
☐	☒	A statement on whether measurements were taken from distinct samples or whether the same sample was measured repeatedly
☐	☒	The statistical test(s) used AND whether they are one- or two-sided<br>*Only common tests should be described solely by name; describe more complex techniques in the Methods section.*
☒	☐	A description of all covariates tested
☐	☒	A description of any assumptions or corrections, such as tests of normality and adjustment for multiple comparisons
☐	☒	A full description of the statistical parameters including central tendency (e.g. means) or other basic estimates (e.g. regression coefficient) AND variation (e.g. standard deviation) or associated estimates of uncertainty (e.g. confidence intervals)
☐	☒	For null hypothesis testing, the test statistic (e.g. *F*, *t*, *r*) with confidence intervals, effect sizes, degrees of freedom and *P* value noted<br>*Give P values as exact values whenever suitable.*
☒	☐	For Bayesian analysis, information on the choice of priors and Markov chain Monte Carlo settings
☒	☐	For hierarchical and complex designs, identification of the appropriate level for tests and full reporting of outcomes
☒	☐	Estimates of effect sizes (e.g. Cohen's *d*, Pearson's *r*), indicating how they were calculated

Our web collection on statistics for biologists contains articles on many of the points above.

Software and code

Policy information about availability of computer code

Data collection	For confocal imaging on Zeiss LSM 880 the ZEN Software 2.3 (black edition) was used. All confocal images were processed in ZEN 2.6 (blue edition). For acquiring FLIM data on the Zeiss LSM 880 system the ZEN software 2.3 (black edition) was used together with the SymPhoTime 64 2.6 software (PicoQuant). FRAP data was collected on a Zeiss LSM 880 using the ZEN Software 2.3 (black edition). For acquiring tr-FAIM data on the Zeiss LSM 880 system the ZEN 2.3 software (black edition) was used together with the SymPhoTime 64 2.6 software (PicoQuant).
Data analysis	FLIM and tr-FAIM data was analyzed using SymPhoTime 64 2.6 software (PicoQuant) and OriginPro 2023b (OriginLab). Two-sided Kolmogorov-Smirnov (KS) statistical test and Spearman correlation were performed using OriginPro 2023b (OriginLab). FRAP analysis was performed using FIJI 1.54f software and Jay_Plugins (https://research.stowers.org/imagejplugins/zipped_plugins.html), Excel (Microsoft Office 365), and Origin Pro 2023b (OriginLab).

For manuscripts utilizing custom algorithms or software that are central to the research but not yet described in published literature, software must be made available to editors and reviewers. We strongly encourage code deposition in a community repository (e.g. GitHub). See the Nature Portfolio guidelines for submitting code & software for further information.

Data

Policy information about availability of data

All manuscripts must include a data availability statement. This statement should provide the following information, where applicable:

- Accession codes, unique identifiers, or web links for publicly available datasets
- A description of any restrictions on data availability
- For clinical datasets or third party data, please ensure that the statement adheres to our policy

The authors confirm that all relevant data are included in this published article (and its supplementary information files). Additional data is available upon request.

Research involving human participants, their data, or biological material

Policy information about studies with human participants or human data. See also policy information about sex, gender (identity/presentation), and sexual orientation and race, ethnicity and racism.

Reporting on sex and gender	n/a
Reporting on race, ethnicity, or other socially relevant groupings	n/a
Population characteristics	n/a
Recruitment	n/a
Ethics oversight	n/a

Note that full information on the approval of the study protocol must also be provided in the manuscript.

Field-specific reporting

Please select the one below that is the best fit for your research. If you are not sure, read the appropriate sections before making your selection.

☒ Life sciences ☐ Behavioural & social sciences ☐ Ecological, evolutionary & environmental sciences

For a reference copy of the document with all sections, see nature.com/documents/nr-reporting-summary-flat.pdf

Life sciences study design

All studies must disclose on these points even when the disclosure is negative.

Sample size	Shown immunostainings and live cell images are representative of at least 2-3 independent experiments. In case of FLIM experiments, at least 30-210 individual cells were recorded and, to ensure reproducibility, experiments were recorded over 2-6 independent days. In case of tr-FAIM experiments, at least 36-45 individual cells were recorded and, to ensure reproducibility, experiments were recorded over 3 independent days. In case of FRAP experiments, at least 45-65 individual junctions were analyzed and, to ensure reproducibility, experiments were recorded over 3-5 independent days. Formal sample size calculation was not performed. Instead, sample size for FLIM experiments was based on previous publications (Austen et al. NCB, 2015; Ringer et al. Nature Methods, 2017; Price et al. Nat Comm, 2019 and Kanoldt et al. Nat Comm, 2020) showing that biological effects can be documented and statistically evaluated with the chosen number of cells. Sample size was kept similar between experimental conditions.
Data exclusions	FLIM, tr-FAIM, and FRAP data was restricted to the cell-cell contact signal as described in Material and Methods. Images with insufficient signal intensity were excluded manually from the analysis. Data exclusion criteria were pre-established.
Replication	All cell-experiments were repeated independently on at least two independent experimental days. Typically, FLIM experiments were repeated over 2-6 days recording 10-20 images on each day per construct and condition. tr-FAIM experiments were repeated over 3 days recording 10-15 images on each day per construct and condition. FRAP experiments were repeated over 3-5 days recording 6-11 movies. All replication attempts were successful and in the expected variability range.
Randomization	In cell-experiments, the groups were determined by the transfected construct and all experiments were started from the same batch of mouse epidermal keratinocytes.
Blinding	For cell data acquisition and analysis the investigators were not blinded, since transiently transfected cells have very different expression levels and cells with expression levels similar to endogenous levels were chosen. Furthermore, the phenotype of cells expressing the different constructs is in many cases obvious and makes blinding impossible.

Reporting for specific materials, systems and methods

We require information from authors about some types of materials, experimental systems and methods used in many studies. Here, indicate whether each material, system or method listed is relevant to your study. If you are not sure if a list item applies to your research, read the appropriate section before selecting a response.

Materials & experimental systems

n/a	Involved in the study
☐	☒ Antibodies
☐	☒ Eukaryotic cell lines
☒	☐ Palaeontology and archaeology
☒	☐ Animals and other organisms
☒	☐ Clinical data
☒	☐ Dual use research of concern
☒	☐ Plants

Methods

n/a	Involved in the study
☒	☐ ChIP-seq
☒	☐ Flow cytometry
☒	☐ MRI-based neuroimaging

Antibodies

Antibodies used

primary antibodies
rabbit anti-α-catenin (Sigma-Aldrich, C2081)
rat anti-E-cadherin (Thermo Fisher Scientific, 13-1900)
rabbit anti-phospho-myosin light chain 2 (Thr18/Ser19) (Cell Signaling, 3674)
mouse anti-tubulin (DM1A) (Sigma-Aldrich, T6199)
mouse anti-vinculin (Sigma-Aldrich, V9131; IF: 1/400)
rat anti-ZO-1 (R26.4C) (Thermo Fisher Scientific, 14-9776-82)

secondary antibodies
anti-mouse IgG Alexa Fluor 405 (Thermo Fisher Scientific, A31553)
anti-mouse IgG Alexa Fluor 488 (Thermo Fisher Scientific, A11001)
anti-mouse IgG HRP (Bio-Rad, 170-6516)
anti-rabbit IgG Alexa Fluor 568 (Thermo Fisher Scientific)
anti-rabbit IgG Alexa Fluor 647 (Thermo Fisher Scientific)
anti-rabbit IgG HRP (Bio-Rad, 170-6515)
anti-rat IgG Alexa Fluor 488 (Thermo Fisher Scientific, A11006)
anti-rat IgG Alexa Fluor 568 (Thermo Fisher Scientific, A11077)

Validation

rabbit anti-α-catenin (polyclonal, Sigma-Aldrich, C2081; IF: 1/1000; WB: 1/7500)
application(s): dot blot: suitable using α-catenin peptide amino acids 890-901 conjugated to BSA; immunohistochemistry (frozen sections): 1:2000 using bovine kidney sections; indirect immunofluorescence: 1:2000 using cultured MDBK cells; microarray: suitable; Western blot: 1:4000 using cultured MDBK cells extract
species reactivity: several mammalian species
specificity: Does not cross-react with β-catenin or γ-catenin (plakoglobin).

rat anti-E-cadherin (monoclonal, Thermo Fisher Scientific, 13-1900; IF: 1/1000)
application(s): Western Blot: 10 µg/mL; Immunohistochemistry; Immunohistochemistry (Paraffin); Immunohistochemistry (PFA fixed); Immunohistochemistry (Frozen): 10 µg/mL; Immunocytochemistry; Flow Cytometry; Immunoprecipitation; Immunomicroscopy
species reactivity: mouse
specificity: This antibody specifically reacts with mouse E-cadherin. This antibody does not inhibit E-cadherin-dependent cell-cell contact.

rabbit anti-phospho-myosin light chain 2 (Thr18/Ser19) (polyclonal, Cell Signaling, 3674; IF: 1/200)
application(s): Western Blotting 1:1000, Simple Western™ 1:10 - 1:50
species reactivity: human, mouse
specificity: Phospho-Myosin Light Chain 2 (Thr18/Ser19) Antibody detects endogenous levels of myosin light chain 2 (smooth muscle) only when dually phosphorylated at threonine 18 and serine 19. The antibody does not cross-react with the cardiac isoform of myosin light chain 2.

mouse anti-tubulin (DM1A) (monoclonal, Sigma-Aldrich, T6199; WB: 1/10000)
application(s): immunocytochemistry: 0.5-1 µg/mL using cultured chicken fibroblasts (CFB); immunohistochemistry: suitable; immunoprecipitation: suitable; microarray: suitable; western blot: 0.5-1 µg/mL using total tissue extract from chicken gizzard
species reactivity: yeast, mouse, amphibian, human, rat, chicken, fungi, bovine
specificity: Anti-α-Tubulin antibody, Mouse monoclonal recognizes an epitope located at the C-terminal end of the α-tubulin isoform (amino acids 426-430) in a variety of organisms (e.g., human, bovine, mouse, and chicken). The antibody is specific for α-tubulin in immunoblotting assays and may be used for localization of α-tubulin in cultured cells or tissue sections. The antibody reacts best with chicken fibroblasts.

mouse anti-vinculin (monoclonal, Sigma-Aldrich, V9131; IF: 1/400)

application(s): immunohistochemistry (frozen sections): suitable; indirect immunofluorescence: 1:400 using cultured human fibroblasts; Western blot: 1:200 using extract of human fibroblasts
species reactivity: bovine, canine, mouse, rat, turkey, human, chicken, frog
specificity: specifically labels vinculin at cell-cell and cell-substrate contacts. Reacts strongly with human vinculin. Shows cross-reactivity with smooth muscle metavinculin.

rat anti-ZO-1 (R26.4C) (monoclonal, Thermo Fisher Scientific, 14-9776-82; IF: 1/100).
application(s): Western Blot; Immunohistochemistry; Immunohistochemistry (PFA fixed); Immunohistochemistry (Frozen): 10 µg/mL; Immunocytochemistry: 1:100
species reactivity: dog, mouse, rat
specificity: The monoclonal antibody R26.4C recognizes the tight junction protein 1, ZO-1 (zona occludens) of rat, mouse, canine, and porcine.

anti-mouse IgG Alexa Fluor 405 (polyclonal, Thermo Fisher Scientific, A31553; IF: 1/200)
application(s): flow cytometry (1-10 µg/ml), immunocytochemistry (1-10 µg/ml), immunofluorescence (1-10 µg/ml), immunohistochemistry (1-10 µg/ml).
species reactivity: mouse

anti-mouse IgG Alexa Fluor 488 (polyclonal, Thermo Fisher Scientific, A11001; IF: 1/500)
application(s): Immunohistochemistry; Immunocytochemistry: 1 µg/mL; Flow Cytometry: 1-10 µg/mL
species reactivity: mouse

anti-mouse IgG HRP (polyclonal, Bio-Rad, 170-6516; WB: 1/10000)
recommended dilution: 1:3000

anti-rabbit IgG Alexa Fluor 568 (polyclonal, Thermo Fisher Scientific, A11036; IF: 1/500)
application(s): immunocytochemistry (4 µg/ml), immunofluorescence (4 µg/ml), immunohistochemistry (1:2000)
species reactivity: rabbit

anti-rabbit IgG Alexa Fluor 647 (polyclonal, Thermo Fisher Scientific, A21244; IF: 1/500)
application(s): Immunocytochemistry: 4 µg/mL; Flow Cytometry: 1-10 µg/mL
species reactivity: rabbit

anti-rabbit IgG HRP (polyclonal, Bio-Rad, 170-6515; WB: 1/10000)
recommended dilution: 1:3000

anti-rat IgG Alexa Fluor 488 (polyclonal, Thermo Fisher Scientific, A11006; IF: 1/500)
application(s): flow cytometry (1-10 µg/ml), immunocytochemistry (4 µg/ml), immunofluorescence (4 µg/ml)
species reactivity: rat

anti-rat IgG Alexa Fluor 568 (polyclonal, Thermo Fisher Scientific, A11077; IF: 1/500)
application(s): Western Blot 1:10,000; Immunohistochemistry (Frozen) 1:1,000; Immunocytochemistry 1-10 µg/mL
species reactivity: rat

Eukaryotic cell lines

Policy information about cell lines and Sex and Gender in Research

Cell line source(s)	AmphoPack 293 cell line (Clonetech - Takara Bio Europe, 631505).<br>Mouse epidermal keratinocytes were a gift from Thomas Magin.<br>MDCK-II cells were a gift from Michael Krahn.
Authentication	The AmphoPack 293 cell line was ordered from the indicated supplier and was not further authenticated. Mouse epidermal keratinocytes and MDCK II cells were not further authenticated.
Mycoplasma contamination	Cells were not tested for mycoplasma contamination.
Commonly misidentified lines<br>(See ICLAC register)	No commonly misidentified cell line was used.

Plants

Seed stocks	n/a
Novel plant genotypes	n/a
Authentication	n/a
